# Violent Recidivism and Adverse Childhood Experiences in Forensic Psychiatric Patients With Impaired Intellectual Functioning

**DOI:** 10.1177/0306624X221133013

**Published:** 2022-11-04

**Authors:** Marija Janković, Geert Van Boxtel, Stefan Bogaerts

**Affiliations:** 1 Tilburg University, The Netherlands; 2 Fivoor Science and Treatment Innovation, Rotterdam, The Netherlands

**Keywords:** Historical-Clinical-Future Revised, dynamic risk factors, dynamic protective factors, violent recidivism, impaired intellectual functioning, adverse childhood experiences

## Abstract

Accurate risk assessment and insight into which factors are associated with recidivism are essential for forensic correctional practice. Therefore, we investigated whether the Historical, Clinical, and Future–Revised (HKT-R [*Historisch Klinisch Toekomst–Revised*]) risk assessment instrument could predict violent recidivism over a 2-year follow-up period in forensic psychiatric patients with intelligence quotient (IQ) < 80. We refer to these patients as intellectually disabled (ID) and patients with IQ ≥ 80 as non-ID. Additionally, the associations of the 14 clinical HKT-R factors with ID versus non-ID group membership were investigated, as well as a possible moderating role of adverse childhood experiences (ACE) in these associations. The final sample encompassed 748 forensic psychiatric patients (15.9% were patients with ID) who were unconditionally released from highly secured Dutch forensic psychiatric institutions between 2004 and 2014. The results showed that the HKT-R total score (AUC = 0.705, 95% confidence interval [CI] [0.527, 0.882]) and the clinical domain (AUC = 0.733, 95% CI [0.579, 0.886]) had a large effect size for predictive validity for 2-year violent recidivism, while the future domain (AUC = 0.653, 95% CI [0.524, 0.781]) and the historical domain (AUC = 0.585, 95% CI [0.397, 0.772]) had a medium effect size for predictive validity for 2-year violent recidivism in ID patients. It was also found that lower levels of self-reliance and social skills were associated with ID, indicating that treatment should prioritize these skills. However, ACE was not associated with ID, nor did it moderate the associations of the clinical HKT-R factors with ID. This study contributes to the understanding of both risk assessment and treatment of forensic psychiatric patients with ID.

Individuals with an intellectual disability (ID) are overrepresented in the criminal justice system and forensic psychiatric facilities (Herrington, 2009). The prevalence of crimes committed by individuals with ID is estimated to be between 2% and 10% and varies depending on the population and methods used (Habets et al., 2015; Lindsay et al., 2011). Research has shown that individuals convicted of a crime who have ID are at greater risk of recidivism than those without ID (Holland & Persson, 2011). According to the *Diagnostic and Statistical Manual of Mental Disorders, 5th Edition* (DSM-5; American Psychiatric Association [APA], 2013), ID is defined as a childhood-onset neurodevelopmental disorder that includes both intellectual (i.e., an intelligence quotient [IQ] of 70 or below) and adaptive functioning deficits in conceptual (e.g., reading and writing ability), social (e.g., communicating with others), and practical (e.g., clothing/bathing one’s self) domains. The DSM-5 has expanded in scope from earlier approaches that relied solely on IQ scores (Schalock et al., 2010), however, IQ is still considered the most representative aspect of ID diagnosis (Wakeling, 2018).

There is ample evidence to suggest that individuals with ID face a number of challenges when encountering the criminal justice system (e.g., Chester et al., 2018). Also, people convicted of a crime who did not meet the DSM-5 ID diagnosis and have an IQ between 70 and 80, can experience overwhelming difficulties in learning and managing everyday living in correctional and forensic settings (Wakeling, 2018). For example, research has shown that they are more likely to break rules in prison, be more often subjected to control and restraint procedures and spend more time in segregation than those with an IQ above 80 (Talbot, 2010). In addition, a large study in a sample of individuals who committed sexual crimes found that those with an IQ below 80 were more likely to have learning difficulties, difficulties with reading, writing, and numeracy, and a lack of work skills than those with an IQ equal to or greater than 80. They also more often had no diploma or permanent residence (Wakeling, 2018). Some of these difficulties may increase the likelihood that patients with ID will reoffend after release from forensic institutions. For example, institutional misconduct has been shown to contribute to all types of recidivism, including violent, property, and other recidivism (Cochran et al., 2014), while finding work and stable housing have been associated with reduced recidivism (Garritsen et al., 2024; Jacobs & Gottlieb, 2020; Ramakers et al., 2017). Taken together, these findings suggest that individuals with IQs between 70 and 80 may also need additional help and support in their daily lives in closed forensic institutions to get the most benefit from their treatment. In this study, we chose a cut-off value of less than 80 to identify forensic psychiatric patients with disabilities in intellectual functioning rather than focusing on patients with an official ID DSM-5 diagnosis. Therefore, we refer to patients with an IQ below 80 as individuals with ID and patients with an IQ of 80 or above as individuals without ID.

In the absence of validated risk assessment tools for individuals with ID, except for the Assessment of Risk Manageability for Intellectually Disabled Individuals Who Offend (ARMIDILO) which was specifically designed for this population (Boer et al., 2004) but not currently available in the Netherlands, forensic clinical staff mostly rely on existing risk instruments developed for individuals without ID (Lindsay & Beail, 2004). Research has confirmed that instruments designed for the mainstream offending population predict recidivism fairly well among the population with ID (e.g., Gray et al, 2007; Lindsay et al., 2008).

One such instrument is the Historical, Clinical, and Risk Management-20 (HCR-20; Webster et al., 1997), which is the most widely used structured professional judgment tool for the assessment of violence risk. It has been shown that the HCR-20 total score significantly predicts violence in individuals who have offended and suffering from ID, with strong performance on the historical scale and somewhat weaker performance on the clinical and risk management scales (Gray et al., 2007). In addition, Lindsay et al. (2008) reported that the HCR-20 has significant discriminatory and predictive validity in high security, medium secure, low security, and community ID services. The Dutch Historical, Clinical, and Future-30 (HKT-30 [*Historisch Klinisch Toekomst–30*]; Comité Instrumentarium Forensische Psychiatrie, 2000) risk assessment instrument, and, its successor, the Historical, Clinical, and Future–Revised (HKT-R [*Historisch Klinisch Toekomst–Revised*]; Spreen et al., 2014) were developed in the Netherlands through a collaboration between scientists and clinicians. The HKT-R is one of the most widely used risk assessment tools in Dutch forensic psychiatry and has been designated by the Ministry of Justice and Security as a mandatory tool for risk assessment and routine outcome monitoring. It was developed to make the assessment more diverse and has a structure *similar* to the HKT-30 and HCR-20. The HKT-R is composed of 12 historical items, 14 clinical items, and 7 future-related items. The predictive validity of the HKT-R in general and violent recidivism was well established in a large heterogeneous sample of forensic psychiatric patients discharged from 1 of 12 highly secured forensic institutions in the Netherlands between 2004 and 2008 (Bogaerts et al., 2018). Results showed that the HKT-R total score (area under the curve [AUC] = 0.78), the historical domain (AUC = 0.75), and the future domain (AUC = 0.71) were modestly predictive, while the clinical domain (AUC = 0.69) was marginally predictive of violent recidivism 2 years after release. AUC values were interpreted as follows: 0.51 to 0.60 low accuracy; 0.60 to 0.70 marginal accuracy; 0.70 to 0.80 modest accuracy; 0.80 to 0.90 moderate accuracy; and over 0.90 high accuracy (Sjöstedt & Grann, 2002). To our knowledge, no previous research has investigated how well the HKT-R performs in predicting violent recidivism among people with ID who offended.

As previously mentioned, a considerable number of studies indicate that the same risk assessments can be used for individuals with and without ID who committed a crime as no significant differences emerged in most risk factor domains (i.e., at scale level) between the two groups (Fitzgerald et al., 2011; Lindsay et al., 2004, 2008). However, the picture is less clear as to whether these two groups differ in the individual risk and protective indicators (i.e., at item level) associated with violent recidivism. For example, by investigating differences in static and dynamic risk factors between young convicted adults with and without ID, Van Der Put et al. (2014) found no significant differences, except for skills. Another study also found that convicted individuals with ID were more likely to have skill problems compared to those without ID (Asscher et al., 2012). In addition, it appears that individuals with ID who have offended have fewer problems with substance use (Asscher et al., 2012). However, they were less likely to accept the responsibility for their offense, had a lower frustration-tolerance threshold, and were more prone to aggressive behavior and impulsivity than those without ID (Asscher et al., 2012; Lindsay & Taylor, 2008; Taggart et al., 2006; Vinkers, 2013). In addition, people with ID convicted of a crime may have difficulty finding stable employment (Vinkers, 2013). These findings suggest that at the level of individual indicators, certain dynamic risk factors are more salient in patients with ID compared to patients without ID. Dynamic risk factors or criminogenic needs are potentially changeable characteristics of individuals and their environments that are expected to increase the likelihood of recidivism, and represent the basis of the *risk* and *need* principles of the Risk-Need-Responsivity model (RNR; Andrews et al., 1990). The RNR is one of the most influential frameworks for the assessment and treatment of offenders. It consists of three principles that should guide the rehabilitation of people convicted of a crime: the *risk principle* implies that the *intensity* and duration of treatment should match the risk level of the individual; the *need principle* states that treatment should target criminogenic needs (i.e., dynamic risk factors); and the *responsivity principle* emphasizes that the intervention should be adapted to the individual’s learning style, motivation, abilities, and strengths (Andrews et al., 1990). That said, treatment is most effective when changing those factors that are crime-related (Andrews & Bonta, 2010). By increasing the understanding of which criminogenic needs are more prevalent and more predictive of recidivism in patients with ID compared to patients without ID, forensic professionals can tailor risk assessment and treatment to the unique needs of convicted individuals with ID. This can lead to more successful reintegration and less recidivism in this group of patients. More research is needed to better understand the criminogenic needs within this population of people with ID convicted of a crime.

Furthermore, individuals with ID are not only at higher risk of being violent toward others but are also often victims of violence themselves (Hassiotis et al., 2019; Keesler, 2020; Kunst et al., 2011). Exposure to adverse childhood experiences (ACE) can be attributed to a combination of characteristics of their disability and impaired family functioning due to that disability (Wigham & Emerson, 2015). ACE refers to potentially stressful or traumatic events that occur during childhood and/or adolescence. They include all forms of abuse and neglect, such as physical abuse, emotional neglect, parental substance use, and exposure to domestic violence. ACE can also refer to traumatic situations, such as living with someone with serious mental illness or losing a parent through divorce, death, or abandonment (Felitti, 2009). If present, it is important to pay attention to traumatic experiences in treatment (Holloway et al., 2018). The literature suggests that ACE is often missed in individuals with ID. This can hinder access to appropriate support (Brewin et al., 2019). Access to clinical interventions for traumatized individuals with ID is highly dependent on appropriate assessment by professionals (Wigham et al., 2021). However, due to their lower cognitive and verbal abilities, individuals with ID do not always understand well what they have been asked or do not recognize their symptoms (Nieuwenhuis et al., 2019). Consequently, ACE is often under-recognized and undertreated in these individuals.

According to neurobiological theories, prolonged exposure to stress in childhood may lead to structural changes in the brain, subsequently causing affective and behavioral dysregulation and cognitive dysfunction (Middlebrooks & Audage, 2008; Van Der Kolk, 2006). For example, IQ scores were found to be eight points lower in children exposed to domestic violence (Koenen et al., 2003). In addition, having ACE increases the chance that the individual will also develop a mental disorder(s), substance use disorders, or both (Jankovic et al., 2021; Messina et al., 2007). In short, these neurological changes induced by ACE may have lasting consequences for emotional regulation, social attachment, and cognitive processing, and may more often lead to the adoption of high-risk behaviors as coping strategies (Anda et al., 2010; Middlebrooks & Audage, 2008).

Although research has shown a strong link between ACE and criminality, it is less clear whether ACE is directly or indirectly associated with future criminal behavior and recidivism (Weber & Lynch, 2021). On the one hand, a substantial body of studies has found that ACE strongly and directly predicts recidivism, but also that those who have committed a previous crime reported higher rates of ACE (e.g., Baglivio et al., 2014, 2015; Wolff et al., 2017). It was argued that these studies reporting a direct association between ACE and recidivism have failed to account for important risk factors in their analysis (Rettinger & Andrews, 2010; Vitopoulos et al., 2019). According to the RNR model, the association between ACE and recidivism is explained by other, well-recognized risk factors for recidivism such as substance use, antisocial peer relationships, and pro-criminal attitudes (Andrews et al., 2006). Evidence-based treatments for convicted offenders usually do not target trauma, but rather focus on reducing risk factors (e.g., antisocial behavior) and increasing protective factors (e.g., coping skills; Abrams & Snyder, 2010; Underwood et al., 2006). Although ACE can predict violent recidivism, it cannot be considered a dynamic risk factor because it cannot be reversed once the individual experienced ACE. In contrast, criminogenic needs are dynamic and can change in two directions (Bonta, 2021). The RNR model also considers the challenges of working therapeutically with individuals who have offended and have experienced traumatic events, as they may have different needs and problems that can affect the responsivity and, consequently, treatment (e.g., Looman & Abracen, 2013). Therefore, ACE can represent a specific responsivity issue (Bonta, 2021). Thus, it is of interest to know better whether the association between ACE and ID still holds when examined alongside the well-established dynamic risk and protective factors for recidivism and how ACE interacts with these risk factors. This could shed some light on understanding whether addressing ACE in individuals with ID who committed a crime could lead to lower recidivism rates after forensic treatment.

The aim of this study is therefore threefold. First, we examine the predictive validity of the HKT-R using a retrospective design to see if it is sensitive in predicting the risk of violent recidivism over a 2-year follow-up period in individuals with ID discharged from highly secure forensic psychiatric institutions. Second, it is investigated how ACE and the individual dynamic risk and protective factors, based on the 14 clinical HKT-R indicators (see Table 1 for an overview), are associated with ID versus non-ID. Lastly, we test if ACE can moderate the associations between the 14 clinical risk and protective HKT-R factors and ID. In the regression models, we control for gender because female forensic patients with ID have more severe victimization histories compared to males (De Vogel & Didden, 2022), while forensic psychiatric patients with ID were more often male than female (Lunsky et al., 2011). With respect to risk and protective factors for violence, the literature suggests no significant differences between women and men (e.g., Strand & Belfrage, 2001). In addition, since no previous research has been done on the predictive validity of the HKT-R in violent recidivism in patients with ID, as well as the association between the clinical HKT-R indicators, ACE and ID, we do not have specific hypotheses about the potential outcomes of this research. Therefore, this study should be considered exploratory. The knowledge generated by this study is relevant to both the assessment (risk principle) and treatment (need and responsivity principles) of forensic psychiatric patients with ID.

**Table 1.** Risk and Protective Factors of the Clinical Domain of the HKT-R.

Risk factors
Antisocial behavior
Hostility
Impulsivity
Violation of terms and agreements
Addiction
Psychotic symptoms
Influence by risky network members
Protective factors
Self-reliance
Treatment cooperation
Labor skills
Social skills
Coping skills
Problem insight
Responsibility for the offense

*Note.* HKT-R = Historical, Clinical, Future–Revised.

## Methods

### Procedure

The demographic, clinical, and criminal data were derived from the archived patient files. These files comprise biographical information, criminal history, psychiatric diagnoses according to the *Diagnostic and Statistical Manual of Mental Disorders* (4th ed., text rev.; *DSM-IV-TR*; APA, 2000), the *IQ* determined by the Dutch Wechsler Adult Intelligence Scale-Fourth Edition (WAIS-IV-NL; Pearson, 2012), treatment progress information, and leave requests. The clinical psychologist administered the WAIS-IV-NL to each patient upon admission to the clinic. It took approximately an hour and a half to complete this test. The split-half reliability of the Full-Scale IQ score was good with an average alpha of α = .97, while the test-retest correlations were also good with a range from *r* *=* .94 to .96 (Pearson, 2012). Moreover, for research purposes, 20 intensively trained psychologists retrospectively coded the HKT-R based on the available file information considering the period of admission to the forensic psychiatric institution. The interrater reliability of the various components of the HKT-R was tested in 60 random files of patients discharged between 2004 and 2008 (Bogaerts et al., 2018; Spreen et al., 2014). The interrater reliability was determined by the intraclass correlation coefficient in a way that values of <.40 are considered as low, values from .40 to .74 as reasonable to good, and values of ≥.75 as very good (Fleiss, 1986). The interrater reliability for the historical scale was .80, for the clinical scale .85 and for the future scale .42. The interrater reliability for the total instrument was .62 (Bogaerts et al., 2018).

Patients were classified into two groups based on their IQ, such that those with an IQ less than 80 belonged to the ID group, while patients with an IQ of 80 or higher belonged to the non-ID group (Wakeling, 2018). Patients were excluded if the files lacked IQ data. In addition, patients without ID were matched on age with patients with ID. All data were anonymized and could not be linked to individual patients. This study was conducted with the exception of the informed consent rule (Article 7:458 paragraph 3 of the Dutch Civil Code [in Dutch: BW]) because it serves the public interest (i.e., the safety of society), and this study with a large group of forensic patients cannot be performed in any other way (as mentioned in Uitzondering op toestemmingsvereiste [Exception to consent requirement; Art. 7: 458 BW]).

The Dutch Ministry and Security of Justice provided official reconviction data on violent recidivism 2 years after release to the researchers for the validation of the HKT-R (Spreen et al., 2014). Forensic psychiatric patients who were released between 2004 and 2008 had been followed from discharge until July 11, 2011, while patients released between 2009 and 2014 had been followed from discharge until June 20, 2018. Ethical permission was given by the Scientific Research Committee of the FPC Kijvelanden, the Dutch Ministry of Security and Justice, the 12 directors of the forensic institutions involved in this study and the Ethical Review Board of Tilburg University.

### Participants

The initial study sample encompassed 815 forensic psychiatric patients who were unconditionally released between 2004 and 2008 (*n* = 347, 8.6% female) and between 2009 and 2014 (*n* = 468, 13.5% female) from any of the 12 Dutch highly secured forensic psychiatric institutions. This means that rules and agreements were no longer imposed and the patients were no longer under the supervision of correctional services. All patients received a TBS [Terbeschikkingstelling] order. TBS, literally translated as “At the disposal of the Government” can be imposed by the criminal court on individuals convicted of a crime with mental health needs who are held not or just partly accountable for their offenses and are considered to stay dangerous for society without treatment (Van Marle, 2000). Forty-nine (6.1%) patients were excluded from this study due to the missing IQ data and additional 18 patients (2.2%) were excluded due to the age-matching procedure. This resulted in a final sample of 748 (10.8% female) patients. Of these, 119 (15.9%) were patients with ID and 629 (84.1%) were patients without ID. The mean age of the patients at the time of admission was 32.73 (*SD* = 9.31, range = 20–79) years and on average, patients stayed in the forensic institution for *9.49 years* (*SD* = 3.82, range = 2–26). Most of the patients were born in the Netherlands (*n* = 575, 76.9%), whereas other patients were born in Suriname (*n* = 44, 5.9%), Curacao (*n* = 30, 4.0%), Morocco (*n* = 23, 3.1 %), Turkey (*n* = 15, 2.0%), or elsewhere (*n* = 61, 8.1%). Prior violent offenses for which the patient received a sentence (including index offenses) were moderate violence (*n* = 390, 52.1%), robbery (*n* = 210, 28.1%), manslaughter (*n* = 170, 22.7%), severe violence (*n* = 164, 21.9%), arson (*n* = 86, 11.5%), sexual violence against adults (*n* = 85, 11.4%), murder (*n* = 63, 8.4%), and sexual violence against minors (*n* = 42, 5.6%). Patients could be convicted of multiple (index) offenses at the same time. At the beginning of treatment, most of the patients were diagnosed with personality disorder not otherwise specified (*n* = 319, 42.6%), followed by substance use disorder (*n* = 275, 36.8%), cluster B personality disorder (*n* = 228, 30.5%), and schizophrenia and other psychotic disorder (*n* = 183, 24.5%). Patients could have more than one mental disorder or illness at the same time. In total, 116 (15.8%) patients recidivated in a new violent crime within 2 years after their release. Patients with ID had a significantly longer treatment duration and generally committed more sexual offenses than patients without ID. There were no other significant differences between the two groups in demographics, criminal history, and psychiatric diagnosis (see Tables 2 and 3). Table 4 presents the means and standard deviations of the ACE and the 14 clinical HKT-R factors. Patients with ID scored significantly lower on problem insight, social skills, and self-reliance than those without ID. No significant between-group differences were detected in ACE and the other clinical HKT-R factors.

**Table 2.** Sample Characteristics.

Variable	Entire sample (*n* = 748)	ID (*n* = 119)	Non-ID (*n* = 629)	
	*M* (*SD*)/*N* (%)			Test statistic
Age at admission	32.73 (9.32)	34.14 (10.54)	32.46 (9.05)	*F*(1, 743) = 3.27
Tretament duration	9.49 (3.82)	10.66 (4.10)	9.27 (3.75)	*F*(1, 738) = 13.39***
Gender (male)	667 (89.2%)	106 (89.1%)	561 (89.2%)	χ^2^(1) = 0.01
Birthland				
Netherlands (yes)	575 (76.9%)	84 (70.6%)	491 (78.1%)	χ^2^(6) = 5.37
Criminal history				
Moderate violence	390 (52.1%)	65 (54.6%)	325 (51.7%)	χ^2^(1) = 0.35
Robbery	210 (28.1%)	30 (25.2%)	180 (28.6%)	χ^2^(1) = 0.58
Manslaughter	170 (22.7%)	27 (22.7%)	143 (22.7%)	χ^2^(1) = 0.00
Severe violence	164 (21.9%)	22 (18.5%)	142 (22.6%)	χ^2^(1) = 0.98
Arson	86 (11.5%)	17 (14.3%)	69 (11.1%)	χ^2^(1) = 1.08
Sexual violence against adults	85 (11.4%)	22 (18.5%)	63 (10.0%)	χ^2^(1) = 7.13**
Murder	63 (8.4%)	5 (4.2%)	58 (9.2%)	χ^2^(1) = 3.27
Sexual violence against minors	42 (5.6%)	13 (10.9%)	29 (4.6%)	χ^2^(1) = 7.13**
Violent recidivism 2 years after release (yes)	116 (15.8%)	13 (11.2%)	103 (16.4%)	χ^2^(1) = 2.21

*Note.* Test statistic refers to the test that was used to evaluate differenecs between patients with and without ID. *n* = number of participants; *SD* = standard deviation; ID = intellectual disability.

**p < .01. ***p < .001.

**Table 3.** Psychiatric Diagnosis.

Variables	Entire sample (*n* = 748)	ID (*n* = 119)	Non-ID (*n* = 629)	
	*N* (%)			Test statistic
Axis I classification				
Psychotic disorders	183 (24.5)	36 (30.3)	147 (23.4)	χ^2^(1) = 2.56
Substance-use disorders	275 (36.8)	46 (38.7)	229 (36.4)	χ^2^(1) = 0.22
Mood disorders	53 (7.1)	5 (4.2)	48 (7.6)	χ^2^(1) = 1.79
Impulse control disorders	22 (2.9)	5 (4.2)	17 (2.7)	χ^2^(1) = 0.79
Dissociative disorders	6 (0.8 )	0 (0.0 )	6 (1.0 )	χ^2^(1) = 1.14
Axis II classification				
Cluster A PDs	23 (3.1)	4 (3.4)	19 (3.0)	χ^2^(1) = 0.04
Cluster B PDs	228 (30.5)	29 (24.4)	199 (31.6)	χ^2^(1) = 2.49
Cluster C PDs	25 (3.3)	4 (3.4)	21 (3.3)	χ^2^(1) = 0.00
PD not otherwise specified	319 (42.6)	44 (37.0)	275 (43.7)	χ^2^(1) = 1.86

*Note.* Test statistic refers to the test that was used to evaluate differenecs between patients with and without ID. *n* = number of participants; ID = intellectual disability; PD = personality disorder. None of the χ^2^ tests were significant.

**Table 4.** Means and Standard Deviations of ACE and Clinical Risk and Protective Factors.

Variables	Entire sample (*n* = 748)	ID (*n* = 119)	Non-ID (*n* = 629)	Test statistic
	*M* (*SD*)			
ACE	2.07 (1.29)	2.12 (1.25)	2.06 (1.30)	*F*(1, 746) = 0.27
Risk factors				
Psychotic symptoms	0.43 (0.78)	0.52 (0.98)	0.41 (0.73)	*U* = 38,636.00
Addiction	0.47 (0.87)	0.38 (0.76)	0.49 (0.89)	*U* = 35,413.00
Impulsivity	1.88 (1.11)	1.97 (1.18)	1.86 (1.10)	*F*(1, 746) = 0.95
Antisocial behavior	1.49 (1.11)	1.49 (1.16)	1.49 (1.10)	*F*(1, 746) = 0.00
Hostility	1.29 (0.92)	1.36 (1.03)	1.28 (0.90)	*F*(1, 746) = 0.80
Violation of terms and agreements	1.13 (1.22)	1.14 (1.32)	1.13 (1.21)	*F*(1, 746) = 0.02
Influence by risky network members	0.92 (1.06)	1.80 (1.17)	0.90 (1.04)	*F*(1, 746) = 1.23
Protective factors				
Problem insight	1.35 (0.86)	1.14 (0.90)	1.39 (0.85)	*F*(1, 746) = 8.24**
Social skills	2.02 (0.81)	1.80 (0.78)	2.06 (0.82)	*F*(1, 746) = 9.89**
Self-reliance	3.42 (0.81)	3.21 (0.97)	3.46 (0.76)	*U* = 31,593.50**
Treatment cooperation	2.51 (1.04)	2.35 (1.08)	2.54 (1.03)	*F*(1, 746) = 3.47
Responsibility for the offense	1.89 (0.96)	1.77 (0.91)	1.92 (0.97)	*F*(1, 746) = 2.31
Coping skills	1.42 (0.83)	1.42 (0.80)	1.42 (0.83)	*F*(1, 746) = 0.00
Labor skills	3.04 (1.03)	2.92 (1.14)	3.07 (1.00)	*F*(1, 746) = 2.10

*Note.* Test statistics refer to the test that was used to evaluate differenecs between patients with and without ID. *n* = number of participants; *SD* = standard deviation; ACE = adverse childhood experiences; ID = intellectual disability.

**p < .05.

### Measures

#### The HKT-R

The HKT-R (Bogaerts et al., 2018; Spreen et al., 2014) is a structured professional risk assessment instrument for assessing the risk of future violent and general recidivism in forensic psychiatric patients after discharge. It consists of 12 historical, 14 clinical, and 7 future risk indicators (see Supplemental Table 1 for the full list of the HKT-R indicators). The historical indicators refer to the patient’s personal history prior to the moment of the offense for which TBS sanctioning was imposed; the clinical indicators refer to the patient’s behavior during the 12 months preceding the risk assessment, and the future indicators refer to the potential risks which could arise after discharge from a forensic psychiatric institution. All indicators are rated on a five-point Likert scale, such that higher scores indicate a higher risk for reoffending (from 0 = *no risk* to 4 = *high risk*). All indicators that belong to the same domain were summed to create a scale score for that domain. The total HKT-R score was created as the sum of all domain-specific scores. The psychometric properties of the HKT-R were tested and validated on 347 former TBS patients who were unconditionally released between 2004 and 2008 from 12 maximum-security forensic psychiatric institutions. The results from this study showed good internal consistency, and good predictive validity of the HKT-R for most forensic target groups (Spreen et al., 2014). In the present study, the internal reliability of the three domains was very good: α = .80 (historical), α = .81 (clinical), and α = .90 (future). The internal reliability of the entire instrument was also very good with Cronbach’s α = .85.

#### ACE and risk and protective factors

The HKT-R (Spreen et al., 2014) was used to assess ACE and dynamic risk and protective factors. In the current study, we used the historical indicator “victim of violence in youth” to assess ACE and the 14 clinical indicators to assess risk and protective factors. Victim of violence in youth assesses whether the patient was the victim of (different) types of assault, maltreatment, and neglect throughout the first 18 years of their life. Maltreatment refers to unacceptable emotional, physical, or sexual behavior toward the patient, while neglect includes physical, emotional, or pedagogical neglect by guardians/caretakers (e.g., being left alone at home at a very young age and bad or irregular meals; Spreen et al., 2014). It was rated on a 5-point Likert scale ranging from 0 = *never a victim of abuse and neglect* to 4 = *chronic neglect and chronic maltreatment.* The 14 clinical indicators were equally divided into seven risk factors (psychotic symptoms, addiction, impulsivity, antisocial behavior, hostility, violation of terms, and influence by risky network members) and seven protective factors (problem insight, treatment cooperation, taking responsibility for the index offense, self-reliance, social skills, coping skills, and labor skills). Dynamic risk and protective factors are potentially changeable aspects of individuals and their environments that are expected to increase (risk factors) or decrease (protective factors) the likelihood of recidivism (Andrews & Bonta, 2010; Heffernan & Ward, 2019). Risk factors were coded such that 0 = *no risk* and 4 = *high risk*, while the original protective factors were reverse-coded, where 0 = *no protection* and 4 = *high protection*.

#### Outcome measures

Violent recidivism was a binary outcome defined as a relapse into criminal behavior within 2 years after discharge, including moderate violence, robbery with violence, serious violence, and arson with the risk for life, (attempted) homicide/murder, and violent sexual assaults on adult victims. It was coded with 0 = *patients who did not recidivate* and 1 = *patients who violently recidivated*. Likewise, ID was used as a binary outcome wherein patients with an IQ ≥ 80 were classified as patients without ID (0) and patients with an IQ < 80 as patients with ID (1).

### Statistical Analysis

All analyses were performed using SPSS Statistics version 24. First, we computed descriptive statistics for all study variables. Differences in sample characteristics between patients with and without ID were evaluated using the Chi-square test for categorical variables and one-way analysis of variance (ANOVA) for normally distributed ordinal or continuous variables. In case of severe violation of the assumption of normality, a non-parametric Mann–Whitney U test was used. Data are normally distributed if absolute values of skewness and kurtosis are not larger than 2 (Field, 2009). In addition, a point-biserial correlation analysis was applied to analyze the associations between ordinal HKT-R indicators, which were treated as continuous in this study, and ID (i.e., the binary outcome variable). It has been stated that ordinal Likert variables with five or more categories can be used as continuous indicators without any harm to the planned analysis (e.g., G. M. Sullivan & Artino, 2013). Missing values on the HKT-R indicators were missing completely at random with χ^2^(5,201) = 5,363.536, *p* = .057 and were therefore replaced by mean.

Furthermore, to test whether the HKT-R can discriminate between patients who did and those who did not violently recidivate, we applied a receiver operating characteristic curve analysis, resulting in the AUC values. To interpret the magnitude of AUC values, effect sizes of 0.56, 0.64, and 0.71 were used as thresholds for low, moderate, and high, respectively, with effect sizes < 0.56 considered to be negligible (Rice & Harris, 2005).

Subsequently, the associations of ACE, and dynamic risk and protective factors with ID were investigated by a means of binary logistic regression. To preserve the statistical power, risk and protective factors were tested separately. The independent variables were ordinal indicators namely ACE, seven risk or seven protective factors, however, they were treated as continuous variables in the binary logistic regression. Before conducting the analysis, the assumptions of the binary logistic regression were checked, including the absence of multicollinearity, the linearity of the continuous variables with respect to the logit of the dependent variable and the independence of observations. In addition, the assumption of a bare minimum of 15, but preferably 20, cases per independent variable must also be met in order to have sufficient statistical power to correctly interpret a significant effect (Hosmer et al., 2013). With a sample of 748 patients, this assumption was met. Furthermore, gender was included as a controlling variable. An interpretation of the results has been made by expressing odds ratios (OR), also known as the exponentiation of the b coefficient [Exp (b)], wherein an OR = 1 means no effect, OR > 1 means that the predictor increases the odds of the outcome, and OR < 1 decreases the odds of the outcome.

Finally, we used PROCESS macro model 1 (Hayes, 2017) to test how the association between the risk or protective factors and ID varies across different levels of ACE (i.e., at the mean and plus/minus one standard deviation from the mean). We tested each factor separately, including gender and the remaining six risk or protective factors as covariates to reveal unique effects.

## Results

The correlations between all study variables are presented in Table 5. ID was weakly and negatively associated with problem insight, social skills, and self-reliance, respectively. ACE and the remaining HKT-R factors were not significantly associated with ID. However, the HKT-R factors correlated significantly with each other (see Table 5 for more detail). Information on skewness and kurtosis of continuous indicators is displayed in Supplemental Table 2.

**Table 5.** Point-Bisserial Correlations Between Predictors and the Binary Outcome.

Variable	1.	2.	3.	4.	5.	6.	7.	8.	9.	10.	11.	12.	13.	14.	15.	16.
1. ACE	1															
2. Psychotic symptoms	- .07	1														
3. Addiction	.07^*^	- .05	1													
4. Impulsivity	.05	.16^**^	.22^**^	1												
5. Antisocial behavior	.12^**^	.18^**^	.15^**^	.46^**^	1											
6. Hostility	.08^*^	.26^**^	.16^**^	.44^**^	.42^**^	1										
7. Violation of terms and agreements	.03	.26^**^	.31^**^	.40^**^	.44^**^	.51^**^	1									
8. Influence by risky network members	.07^*^	.09^*^	.05	- .01	.16^**^	.01	.14^**^	1								
9. Problem insight	- .03	- .21^**^	.03	- .15^**^	- .30^**^	- .21^**^	- .24^**^	- .18^**^	1							
10. Social skills	- .12^**^	- .09^*^	- .11^**^	- .32^**^	- .40^**^	- .35^**^	- .33^**^	- .10^**^	.18^**^	1						
11. Self-reliance	.03	- .30^**^	.03	- .12^**^	- .14^**^	- .13^**^	- .14^**^	- .08^*^	.15^**^	.22^**^	1					
12. Treatment cooperation	.03	- .21^**^	- .14^**^	- .29^**^	- .45^**^	- .40^**^	- .42^**^	- .16^**^	.48^**^	.31^**^	.26^**^	1				
13. Responsibility for the offense	- .05	- .24^**^	.01	- .13^**^	- .27^**^	- .18^**^	- .20^**^	- .12^**^	.46^**^	.11^**^	.07	.35^**^	1			
14. Coping skills	- .13^**^	- .15^**^	- .13^**^	- .47^**^	- .41^**^	- .38^**^	- .35^**^	- .02	.23^**^	.40^**^	.13^**^	.36^**^	.20^**^	1		
15. Labor skills	- .05	- .24^**^	- .13^**^	- .26^**^	- .34^**^	- .27^**^	- .32^**^	- .08^*^	.21^**^	.33^**^	.37^**^	.43^**^	.22^**^	.33^**^	1	
16. ID patients	.02	.05	- .04	.04	.00	.03	.00	.04	- .11^**^	- .11^**^	- .12^**^	- .07	- .06	.00	- .05	1

*Note*. *p < .05. **p < .01. ACEs = adverse childhood experiences; ID = intellectual disability.

Furthermore, the predictive validity of the different domains of the HKT-R for violent recidivism within 2 years after release was tested in a sample of forensic psychiatric patients with ID. Results showed that the HKT-R total score (AUC = 0.705, 95% confidence interval [CI] [0.527, 0.882]) as well as the clinical domain (AUC = 0.733, 95% CI [0.579, 0.886]) were highly predictive of 2-year violent recidivism. In contrast, the future domain (AUC = 0.653, 95% CI [0.524, 0.781]) and the historical domain were moderately predictive (AUC = 0.585, 95% CI [0.397, 0.772]) of 2-year violent recidivism.

Subsequently, the associations of ACE, and dynamic risk and protective factors with ID were investigated with binary logistic regression. First, we checked whether the data met the assumptions. We did not detect multicollinearity amongst independent variables. In addition, all independent variables were linearly related to the logit of the dependent variable, as determined by the Box-Tidwell test (Box & Tidwell, 1962). Since all assumptions were met, we proceeded with the analysis. It was first investigated how ACE and seven risk factors were associated with ID, controlled for gender. Results (see Supplemental Table 3) showed that the logistic model was not statistically significant χ^2^(9) = 6.972, *p* = .640. In addition, ACE, seven risk factors, and gender did not contribute significantly to the model. This means that ACE, risk factors, and gender are not differentially associated with ID compared to non-ID.

Moreover, we repeated the analysis but this time with the seven protective factors entered into the model as independent predictors, along with ACE and gender. The model was statistically significant, χ^2^(9) = 24.047, *p* = .004. It explained 5.4% (Nagelkerke *R*^2^) of the variance in the ID and correctly classified 84.1% of cases. Lower self-reliance (OR = 0.77) and lower social skills (OR = 0.67) were significantly associated with ID group membership. The remaining five protective factors and ACE did not contribute significantly to the model (see Table 6) and gender was not a significant covariate either.

**Table 6.** Logistic Regression Model With Protective Factors, ACE, and Intellectual Disability.

	*b*	*SE*	*p*-Value	Exp(*b*)	95% CI for Exp(*b*)	
					Lower	Upper
ACE	0.037	0.080	.641	1.038	0.887	1.214
Problem insight	−0.286	0.150	.056	0.751	0.560	1.008
Social skills	−0.401	0.147	.006	0.670	0.502	.894
Self-reliance	−0.258	0.122	.035	0.772	0.608	.981
Treatment cooperation	0.014	0.124	.911	1.014	0.796	1.292
Crime responsibility	−0.056	0.125	.651	0.945	0.740	1.207
Coping skills	0.255	0.140	.069	1.290	0.981	1.697
Labor skills	0.041	0.114	.718	1.042	0.833	1.304
Gender	0.071	0.331	.830	1.074	0.561	2.055
Constant	−0.237	0.598	.692	0.789		

*Note*. ACE = adverse childhood experiences; *SE* = standard error; CI = confidence intervals.

Lastly, to investigate whether ACE moderates the associations of risk and protective factors with ID, a moderation analysis was conducted. However, ACE was not a significant moderator in these associations. The results are presented in Supplemental Table S4.

## Discussion

In this study, we investigated the predictive validity of the HKT-R risk assessment tool for violent recidivism 2 years after release among forensic psychiatric patients with ID. In addition, the associations of dynamic risk and protective factors with ID versus non-ID group membership were investigated, as well as a potential moderating role of ACE in these associations. The study was conducted among a representative sample of forensic psychiatric patients who were released unconditionally between 2004 and 2014 from 12 highly secured forensic psychiatric institutions in the Netherlands.

We found that the predictive validity of both the HKT-R total score and the clinical domain was high for 2-year violent recidivism, while the future domain and the historical domain were moderately predictive for 2-year violent recidivism. These findings are to a large extent parallel to the findings obtained in a heterogeneous sample of forensic psychiatric patients (Bogaerts et al., 2018). The only difference concerns the historical and clinical domains, with the former doing slightly worse and the latter doing slightly better in the subsample of forensic psychiatric patients with ID. Our finding is in line with previous studies indicating that risk assessment instruments designed for the mainstream forensic psychiatric population, can also discriminate fairly well between people with ID who violently recidivated and those who did not (Gray et al, 2007, Lindsay et al., 2008). In other words, the HKT-R can be reliably used to support forensic psychiatric professionals in their clinical decisions to estimate future violent recidivism in patients with ID. In addition, the clinical domain of the HKT-R had particularly high predictive validity for violent recidivism in the ID population. This corresponds with the need principle of the RNR model (Andrews et al., 1990) which states that to reduce recidivism, treatment should target dynamic (i.e., clinical) risk factors. However, in this study, we did not investigate how changes in these dynamic risk factors are associated with the likelihood of recidivism after release, which could be useful in future research. Finally, our study supports the use of the HKT-R clinical indicators for Routine Outcome Monitoring (ROM-)measurements to evaluate the forensic treatment of patients with ID (Spreen et al., 2014).

We also found that lower self-reliance and lower social skills were significantly associated with ID group membership, while ACE and the other clinical HKT-R factors were not significantly associated with ID, controlled for gender. This means that forensic psychiatric patients who were less able to perform essential daily tasks independently (e.g., personal hygiene, dealing with money, and sleeping patterns) and patients who were less able to maintain social contact with others in an acceptable and successful way were more likely to belong to the ID group than to non-ID. This is in line with previous studies demonstrating that people with ID convicted of a crime have more problems with skills necessary to perform daily activities and cope with social interactions than those without ID (Asscher et al., 2012; Hall, 2000; Smith et al., 2015; Van Der Put et al., 2014). Notably, a lack of social skills can seriously restrict the possibility of maintaining intimacy giving rise to sexual offending. Indeed, in the current study, we found that patients with ID committed more sexual offenses than patients without ID. This finding is consistent with several previous studies demonstrating that sexual offenses are more common among individuals with ID (e.g., Ray et al., 2019; Simpson & Hogg, 2001). Another factor that can also contribute to (sexual) offending in individuals with ID is the lack of knowledge about what behavior is appropriate (Eastgate, 2008). However, in this study, problem insight, which was defined as awareness of risk factors and signals of risky behavior in risky situations (Spreen et al., 2014), was not significantly associated with ID group memberships when entered into the model together with ACE and other protective factors. Nonetheless, problem insight appeared to have a significant positive bivariate association with ID. Problem insight may not be significantly associated with ID due to the shared variance with the other protective factors, especially treatment cooperation and responsibility for the offense.

Furthermore, the finding that the other risk and protective factors were not significantly associated with ID is consistent with a large body of studies that also found no significant differences between the two groups in most risk domains (Fitzgerald et al., 2011; Lindsay et al., 2004, 2008). The current study adds to the literature by demonstrating that there are no significant differences between patients with and without ID in most individual risk and protective factors. Alternatively, it could be that the power to obtain statistically significant effects in this study was somewhat lower because the number of patients without ID was about five times higher than the number of patients with ID. Yet, some studies found that people with ID convicted of a crime have fewer problems with substance use, lower tolerance for frustration, less employment opportunities, and are more prone to impulsiveness than those without ID (Asscher et al., 2012; Lindsay & Taylor, 2008; Taggart et al., 2006; Vinkers, 2013). These inconsistencies with previous research can be attributed to the characteristics of a forensic psychiatric sample. In other words, most of these previous studies investigated differences in risk factors between individuals with justice system involvement and people convicted of a crime with mental health needs, however, our study concerns the forensic psychiatric patients with and without ID. That said, patients without ID in our sample have other psychiatric diagnoses that may affect risk or protective factors in a similar way, except for self-reliance and social skills. Therefore, increasing skills and tailoring treatment to the level of intellectual functioning may be an important aspect of improving the reintegration of forensic psychiatric patients with ID (Asscher et al., 2012).

Contrary to the findings of previous studies (Keesler, 2020; P. M. Sullivan & Knutson, 2000), we found no direct effect of ACE on ID. This indicates that forensic psychiatric patients who experienced more ACE have an equal chance of belonging to the ID group as those who experienced no or less ACE. The finding can again be explained by the characteristics of a forensic psychiatric sample. ACE is highly prevalent in forensic psychiatric patients. For example, Karatzias et al. (2019) reported the ACE prevalence of 79.2% in a sample of Scottish forensic patients residing in one of the high, medium, or low security sites. In our study, this prevalence was even higher with 84.3% of patients experiencing at least one type of childhood adversity. Therefore, it might be that we fail to find a significant direct effect due to the homogeneity of our sample in terms of ACE. Likewise, ACE was found not to be a significant moderator, suggesting that there are no differences in the risk and protective factors between patients with ID and more ACE and patients with ID and less or no ACE. This important finding indicates that ACE, at least in our study group, should not be considered a criminogenic need in offender rehabilitation (Bonta, 2021). However, personal reactions to ACEs can greatly influence the development of a case management plan and how the treatment is delivered. Therefore, these responses may need to be addressed before targeting criminogenic needs in treatment (Bonta, 2021). Similarly, Andrews et al. (1990, 2006) state that ACE should not be targeted in the treatment as a criminogenic need because it does not predict recidivism directly, but only indirectly through substance use, antisocial peers, and antisocial attitudes, among others. Further research is needed to clarify the role of ACE on recidivism in a presence of other well-established criminogenic needs.

This study is not without limitations and the findings should be interpreted with caution. One obvious limitation is that the patients with ID were disproportionately represented in this sample compared to the patients without ID, which could negatively affect the statistical power to find significant effects. As such, further research may attempt to obtain more equal sample sizes in each group. Another limitation is that we have used cumulative ACE to describe all types of abuse, however, the findings cannot speak to the differential impact of various forms of ACE on ID. For example, a literature review showed that individuals with ID are at a higher risk of sexual abuse than those without ID (Byrne, 2018). It is, therefore, possible that this specific type of abuse rather than a cumulative ACE score influences risk and protective factors in individuals with ID. Future research may want to explore how different forms of ACE, particularly sexual abuse, are associated with ID. It may also investigate the incremental validity of ACE beyond the risk and protective factors for violent recidivism prediction. The study was also limited because it did not take into account possible gender differences when investigating the predictive validity of the HKT-R. Given the evidence that ID is more common in male forensic patients (Lunsky et al., 2011), the HKT-R may not be equally valid in the prediction of violent recidivism for male and female forensic patients. However, due to the small sample size of women, we were unable to investigate the predictive validity of the HKT-R separately for men and women. This would be important to address in future research. Finally, the findings of the present study may not be generalizable to other international forensic samples due to differences in sentencing. For example, in the United States, patients with personality disorders and/or substance use disorders would likely have been sent to prison rather than to forensic psychiatric institutions (De Ruiter & Trestman, 2007). Since the HKT-R is comparable with the internationally used HCR-20 to assess an individual’s risk of violence, our findings may apply to other countries with similar sentencing options for individuals who have committed crimes under the influence of severe mental illness.

In conclusion, the present study showed that the HKT-R tool developed to estimate violent recidivism in a diverse sample of forensic psychiatric patients performs equally well in a subsample of forensic psychiatric patients with ID. In addition, we also found no significant differences in most individual risk and protective factors between patients with and without ID. This supports the claim that it is justified to use the HKT-R for risk assessment in patients with ID detained in correctional clinics. Nonetheless, we did find that lower levels of self-reliance and lower levels of social skills were significantly associated with ID group membership, meaning that treatment should pay attention to these skills. In contrast, ACE was not associated with ID, nor did it moderate the associations of risk and protective factors with ID. That said, targeting ACE in the rehabilitation of forensic patients with ID would probably not influence the risks associated with violent recidivism. However, incorporating trauma-informed care in forensic treatment, and particularly in the treatment of individuals with ID, may have beneficial effects on the health and well-being of patients. For example, eye movement desensitization and reprocessing therapy have been shown to improve mood, reduce physical complaints, enhance the acquisition of new skills, and increase self-reliance in individuals with ID (e.g., Nieuwenhuis et al., 2019). ACE may be better considered as a responsivity factor than as a risk factor for recidivism in forensic patients with ID. Therefore, patients with ID and ACE may need more treatment adjustments. This study provides valuable information for both risk assessment and treatment of forensic psychiatric patients with ID.

## Supplemental Material

sj-docx-1-ijo-10.1177_0306624X221133013 – Supplemental material for Violent Recidivism and Adverse Childhood Experiences in Forensic Psychiatric Patients With Impaired Intellectual Functioning Supplemental material, sj-docx-1-ijo-10.1177_0306624X221133013 for Violent Recidivism and Adverse Childhood Experiences in Forensic Psychiatric Patients With Impaired Intellectual Functioning by Marija Janković, Geert Van Boxtel and Stefan Bogaerts in International Journal of Offender Therapy and Comparative Criminology
