# Supplementary material for: Violent Recidivism and Adverse Childhood Experiences in Forensic Psychiatric Patients With Impaired Intellectual Functioning
Source: Int J Offender Ther Comp Criminol. 2022 Nov 4;68(13-14):1357–79. doi: 10.1177/0306624X221133013 (PMC11375903; doi:10.1177/0306624X221133013)
Supplement: sj-docx-1-ijo-10.1177_0306624X221133013 – Supplemental material for Violent Recidivism and Adverse Childhood Experiences in Forensic Psychiatric Patients With Impaired Intellectual Functioning [file sj-docx-1-ijo-10.1177_0306624X221133013.docx]

**Supplementary Materials**

**Table S1**

The Historical Clinical Future *[Historisch Klinisch Toekomst]* Revised Risk Assessment *Instrument (Spreen et al., 2014)*

| The Historical domain (H domain) | The Clinical domain (K domain) | The Prospective domain (T domain) |
| --- | --- | --- |
| H01 Judicial History | K01 Problem insight | T01 Concurrence on arrangements regarding crime-prevention |
| H02 Breach of conditions for treatment and supervision | K02 Psychotic symptoms | T02 Living arrangements |
| H03 Age first offence | K03 Addiction | T03 Finances |
| H04 Victim type | K04 Impulsivity | T04 Work |
| H05 Network influence | K05 Antisocial behavior | T05 Leisure Time |
| H06 Behavioral issues before the age of 12 | K06 Hostility | T06 Social Network |
| H07 Victim of violence in youth (before 18 years of age) | K07 Social Skills | T07 Stressful circumstances |
| H08 Care History | K08 Self-reliance |  |
| H09 Employment history | K09 Cooperation with treatment |  |
| H10 Addiction history | K10 Responsibility for the offense |  |
| H11 Instability related to living arrangements | K11 Coping Skills |  |
| H12 Instability related to finances | K12 Violation of terms and agreements |  |
|  | K13 Labor skills |  |
|  | K14 Influence of protective and/or risky network-members |  |

**Table S2**

*Skewness and Kurtosis*

|  | Minimum | Maximum | Median | Skewness | Kurtosis | *SE* |
| --- | --- | --- | --- | --- | --- | --- |
| Age at admission | 20 | 79 | 31 | 0.91 | 0.90 | 0.34 |
| Treatment duration | 2 | 26 | 9 | 0.95 | 0.92 | 0.14 |
| ACEs | 0 | 4 | 2 | - 0.01 | - 0.89 | 0.05 |
| Psychotic symptoms | 0 | 4 | 0 | 2.91 | 9.21 | 0.03 |
| Addiction | 0 | 4 | 0 | 2.36 | 4.93 | 0.03 |
| Impulsivity | 0 | 4 | 1.88 | - 0.14 | - 0.44 | 0.04 |
| Antisocial behavior | 0 | 4 | 1.49 | 0.37 | - 0.43 | 0.04 |
| Hostility | 0 | 4 | 1.29 | 0.49 | 0.22 | 0.03 |
| Violation of terms and agreements | 0 | 4 | 1.13 | 1.00 | 0.06 | 0.04 |
| Influence by risky network members | 0 | 4 | 0.92 | 1.18 | 0.49 | 0.04 |
| Problem insight | 0 | 4 | 1.35 | 0.51 | 0.43 | 0.03 |
| Social skills | 0 | 4 | 2.02 | 0.24 | 0.76 | 0.03 |
| Self-reliance | 0 | 4 | 3.42 | - 1.84 | 3.19 | 0.03 |
| Treatment cooperation | 0 | 4 | 2.51 | - 0.31 | - 0.25 | 0.04 |
| Responsibility for the offense | 0 | 4 | 1.89 | 0.54 | 0.45 | 0.04 |
| Coping skills | 0 | 4 | 1.42 | 0.58 | 1.31 | 0.03 |
| Labor skills | 0 | 4 | 3.04 | - 1.41 | 1.75 | 0.04 |

*Note*. ACE = Adverse childhood experiences; *SE* = Standard error.

**Table S3**

*Logistic Regression Model With Risk Factors, ACE and Intellectual Disability*

|  | *b* | *S.E.* | *p* | *Exp(b)* | *95% CI* for *Exp(b)* | |
| --- | --- | --- | --- | --- | --- | --- |
|  |  |  |  |  | Lower | Upper |
| ACE | .047 | .079 | .555 | 1.048 | .897 | 1.224 |
| Psychotic symptoms | .134 | .126 | .286 | 1.144 | .894 | 1.464 |
| Addiction | - .173 | .136 | .204 | .841 | .644 | 1.099 |
| Impulsivity | .126 | .111 | .257 | 1.134 | .912 | 1.411 |
| Antisocial behavior | - .093 | .110 | .399 | .911 | .734 | 1.131 |
| Hostility | .082 | .137 | .549 | 1.085 | .830 | 1.419 |
| Violation of terms and agrrements | - .038 | .106 | .723 | .963 | .782 | 1.186 |
| Influence by risky network members | .114 | .094 | .222 | 1.121 | .933 | 1.347 |
| Gender | - .038 | .325 | .907 | .963 | .509 | 1.822 |
| Constant | - 1.985 | .442 | .000 | .137 |  |  |

*Note*. ACE = Adverse childhood experiences; *S.E.* = Standard error; *CI* = Confidence intervals.

**Table S4**

*Interactions Between the HKT-R Clinical Factors and ACE in Predicting ID*

|  | *b* | *S.E.* | z | *p* | *95% CI* for *Exp(b)* | |
| --- | --- | --- | --- | --- | --- | --- |
|  |  |  |  |  | Lower | Upper |
| Risk factors |  |  |  |  |  |  |
| Psychotic symptoms x ACE | .090 | .086 | 1.049 | .294 | -.078 | .259 |
| Addiction x ACE | .010 | .102 | .100 | .920 | -.189 | .209 |
| Impulsivity x ACE | .070 | .071 | .987 | .324 | -.069 | .210 |
| Antisocial behavior x ACE | -.039 | .070 | -.557 | .578 | -.177 | .099 |
| Hostility x ACE | .148 | .078 | 1.892 | .059 | -.005 | .301 |
| Violation of terms and agrrements x ACE | -.042 | .063 | -.661 | .508 | -.165 | .082 |
| Influence by risky network members x ACE | -.019 | .069 | -.280 | .779 | -.154 | .116 |
| Protective factors |  |  |  |  |  |  |
| Problem insight x ACE | -.030 | .097 | -.310 | .757 | -.219 | .159 |
| Social skills x ACE | -.064 | .099 | -.642 | .521 | -.258 | .131 |
| Self-reliance x ACE | -.140 | .086 | -1.630 | .103 | -.309 | .028 |
| Treatment cooperation x ACE | -.089 | .076 | -1.164 | .244 | -.239 | .061 |
| Crime responsibility x ACE | .000 | .086 | .005 | .996 | -.168 | .169 |
| Coping skills x ACE | -.107 | .090 | -1.182 | .237 | -.283 | .070 |
| Labor skills x ACE | -.029 | .079 | -.364 | .716 | -.183 | .126 |

*Note*. ACE = Adverse childhood experiences; ID = Intellectual disability; *S.E.* = Standard error; *CI* = Confidence intervals.
